# Neonatal screening for congenital adrenal hyperplasia in Southern Brazil: a population based study with 108,409 infants

**DOI:** 10.1186/s12887-016-0772-x

**Published:** 2017-01-17

**Authors:** Cristiane Kopacek, Simone Martins de Castro, Mayara Jorgens Prado, Claudia Maria Dornelles da Silva, Luciana Amorim Beltrão, Poli Mara Spritzer

**Affiliations:** 1 Neonatal Screening Labor, Neonatal Screening Unit, Hospital Materno Infantil Presidente Vargas, Porto Alegre, RS Brazil; 2 Departamento de Análises, School of Pharmacy, Universidade Federal do Rio Grande do Sul, Porto Alegre, RS Brazil; 3 Fundação Estadual de Projetos de Pesquisa em Saúde (FEPPS), Porto Alegre, RS Brazil; 4 Gynecological Endocrinology Unit, Division of Endocrinology, Hospital de Clinicas de Porto Alegre, Universidade Federal do Rio Grande do Sul, Porto Alegre, RS Brazil; 5 Faculdade de Farmácia–UFRGS, Av. Ipiranga, 2752, Porto Alegre, RS 90610-000 Brazil

**Keywords:** Congenital adrenal hyperplasia, Incidence, Neonatal screening, Mass screening, 21-amino-17-hydroxyprogesterone

## Abstract

**Background:**

Congenital adrenal hyperplasia (CAH) is an autosomal recessive disorder associated with inborn errors of steroid metabolism. 21-hydroxylase enzyme deficiency occurs in 90 to 95% of all cases of CAH, with accumulation of 17 hydroxyprogesterone (17-OHP). Early diagnosis of CAH based on newborn screening is possible before the development of symptoms and allows proper treatment, correct sex assignment, and reduced mortality rates. This study describes the results obtained in the first year of a public CAH screening program in the state of Rio Grande do Sul, Brazil.

**Methods:**

We reviewed the screening database in search of babies with suspected CAH, that is, altered birth-weight adjusted 17-OHP values at screening. The following data were analyzed for this population: screening 17-OHP values, retest 17-OHP values, serum 17-OHP values for those with confirmed CAH on retest, maternal and newborn data, and family history of CAH. For the screening program, 17-OHP levels are determined on dried blood spots obtained in filter paper with GSP solid phase time-resolved immunofluorescence.

**Results:**

Of 108,409 newborns screened, eight were diagnosed with CAH (four males, four females). The incidence of CAH in the state was 1:13,551. Six cases were identified as classic salt-wasting CAH and two were cases of virilizing CAH. The positive predictive value (PPV) of the initial screening (before diagnostic confirmation) was 1.6%. The overall rate of false positive results was 0.47%. The number of false positive results was higher among newborns with birth weight < 2000 g.

**Conclusion:**

The present results support the need for CAH screening by the public health care system in the state, and show that the strategy adopted is adequate. PPV and false positive results were similar to those reported for other states of Brazil with similar ethnic backgrounds.

## Background

Congenital adrenal hyperplasia (CAH) is an autosomal recessive disorder associated with inborn errors of steroid metabolism caused by deficiency of enzymes involved in the biosynthesis of cortisol from cholesterol [1]. 21-hydroxylase deficiency occurs in 90 to 95% of all cases of CAH and is related to mutations in the CYP21A2 gene [1, 2]. In the presence of 21-hydroxylase deficiency, 17 hydroxyprogesterone (17-OHP) accumulates and is diverted to androgen synthesis with virilizing effects [1, 2]. Mineralocorticoid synthesis may or may not be reduced, depending on the extent to which 21-hydroxylase activity is impaired [1, 3].

Three clinical forms of CAH have been recognized: two classic forms, salt-wasting CAH (SW) and simple virilizing CAH (SV), and non-classic, late onset CAH (NC). SW is the most prevalent, occurring in around 75% of newborns with a diagnosis of CAH (1). Considering that the salt loss crisis is critical and starts in the second week of life, early diagnosis of classic forms of CAH based on newborn screening is desirable even before the beginning of symptoms. This allows proper treatment, correct sex assignment, and reduced mortality rates [2, 4, 5]. CAH occurs in about one of every 10,000 to 18,000 live births in the general population, and is more common in Caucasians [1]. Incidence varies according to ethnicity and geographical region [1, 6]. In addition, 17-OHP levels in neonates are affected by factors such as gestational age at birth, birth weight, and age at the time of 17-OHP testing [7–11]. Perinatal stress has been associated with high values of 17-OHP on screening [8, 12], while maternal use of corticosteroids towards the end of pregnancy and early sample collection seem to reduce these values [10, 13]. Reference 17-OHP values for diagnosis of CAH in full term newborns vary from 15 to 40 ng/mL among different laboratories.

Because of the many factors impacting the outcome of CAH screening, the stratification of 17-OHP values according to birth weight is recommended in order to decrease false positive results [8, 10, 14]. A high rate of false positive results translates into increased health care cost and distress for families [15–17].

Even though screening for CAH has been available through the public health care system for many years in some Brazilian states [10, 16, 18, 19], only in May 2014 was it introduced in the southernmost state of Rio Grande do Sul. Therefore, the aims of the present study were to summarize the results of the first year of CAH newborn screening in this population, to determine the incidence of CAH in the state, and to estimate the rate of false positive results in the local screening program.

## Methods

### Design and population

A population-based study was conducted with newborns included in the first year of a public CAH screening program in the state of Rio Grande do Sul, Brazil (May 2014 to April 2015). For the screening program, dried blood samples (heel prick test) are collected 2 to 40 days after birth. Babies with positive screening are retested. Participation is open to public and private primary care facilities, health care units, hospitals, and maternity hospitals. The study population corresponded to about three-fourths of the live newborns in the state during this period. The other 25% of newborn babies are screened in private outpatient services, and data from this population are not freely available.

**Fig. 1:**
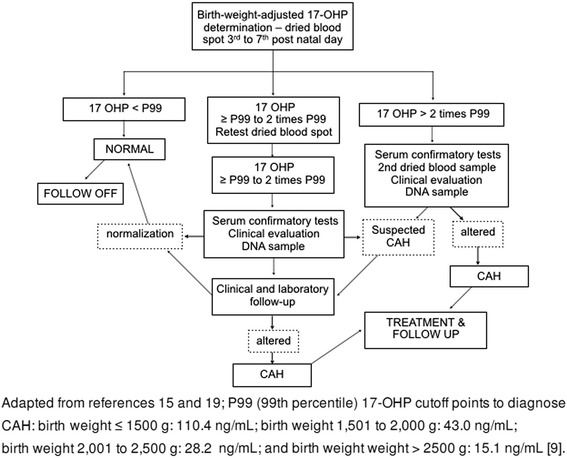
Flow diagram of newborn screening for congenital adrenal hyperplasia

In the present study, we reviewed the screening database in search of babies with suspected CAH, that is, altered 17-OHP values at screening. The following data were analyzed for this population: screening 17-OHP values, retest 17-OHP values, serum 17-OHP values (for those with suspected CAH on screening and retest), maternal and newborn data, and family history of CAH. Figure 1 describes the screening strategy.

The study protocol was approved by the Research Ethics Committee at Hospital Materno Infantil Presidente Vargas, and meets the guidelines and norms regulating research involving human beings.

### Blood collection and 17-OHP measurements

Dried blood spots were obtained using filter paper (S & S 903). 17-OHP was measured with the GSP solid phase [time-resolved] immunofluorescence assay (Neonatal 17-OHP kit–PerkinElmer, Turku, Finland). The linearity range for serum 17-OHP concentration was 0.9 to 229 ng/mL.

The reference 17-OHP values used in the present study are those recommended by the Brazilian National CAH Screening Program [20], which were based on a pilot study with the population of the state of São Paulo [10]. Four birth weight tiers were established: tier 1, birth weight ≤ 1500 g; tier 2, birth weight 1501 to 2000 g; tier 3, birth weight 2001 to 2500 g; and tier 4, birth weight > 2500 g. For each tier, the 99th percentile (P99) 17-OHP cut-off levels to diagnose CAH were 110.4, 43.0, 28.2 and 15.1 ng/mL respectively. In the pilot study, newborns from mothers with informed corticosteroid use late in pregnancy were called for a second collection after 15 days of life. This record was added to the filter paper in order to minimize the risk of false negative results [13]. For the present study, early (<48 h) samples collected for 17-OHP determinations were excluded. In the Rio Grande do Sul screening program, CAH screening is based on samples collected between the 2nd and 40th post-natal days. Samples from 0 to 1 days and/or without weight information were excluded from this analysis, but these babies were called for immediate new collection in the valid period and with correct weight information.

Classic CAH (SW and SV) was diagnosed by increased 17-OHP on screening, confirmed by dried blood spot retest and further clinical evaluation showing virilized external genitalia in girls and salt-wasting signs in both sexes and serum/dried blood spot 17-OHP measurement.

### Statistical analysis

Descriptive data were expressed as mean ± standard deviation (SD) or median and 25–75 interquartile range. Categorical variables are reported as frequencies (%). Log10 transformation was used to normalize the distribution of non-Gaussian variables and Student’s t test was used for comparisons between two groups. Categorical variables were compared using Fisher’s exact test. Generalized estimating equations (GEE) were used to estimate the interaction between birth weight tier and the difference (delta) between 17-OHP levels at screening and retest, followed by Bonferroni test. All analyses were performed using the Statistical Package for the Social Sciences 22.0 (SPSS, Armonk, NY, USA). Data were considered to be significant at *p* < 0.05.

## Results

Of the 108,409 total samples obtained at the initial screening, 104,737 were collected between the 3rd and 40th post-natal days, and included in the present analysis, corresponding to 98.4% of the total. Of these, 83,424 (77%) were collected at age 3–7 days. Most retest samples were collected around the second or third week of life [median 17 (14.0–21.0) days]. Eight newborns were diagnosed with CAH (four males, four females). None of the four females had a clinical diagnosis of CAH prior to the screening: the first female presented genital ambiguity of unknown etiology; the second was initially considered as a male; and in the other two females, clitoromegaly was not recognized. Two deaths occurred, one due to complications associated with several malformations and the other due to hyponatremia and metabolic acidosis. In this child, screening was not performed until 38 days of life.

**Fig. 2:**
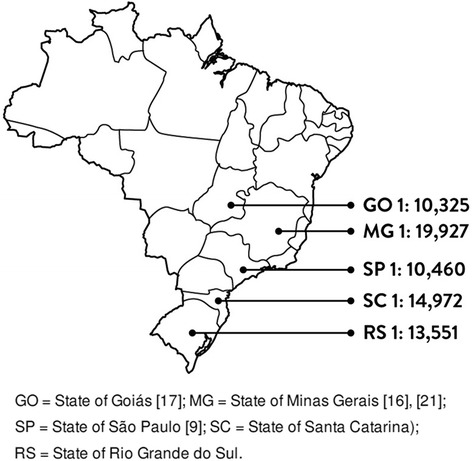
Reported incidence of CAH at neonatal screening in different states of Brazil

The incidence of CAH in the state was 1:13,551. Six cases were identified as classic salt-wasting CAH and two were cases of virilizing CAH. Figure 2 shows the incidence of CAH in the state and in the other Brazilian states.

During this first year, 514 infants (0.47% of the total screened population) had 17-OHP levels that were higher than the reference cut-off levels (>P99 or two times P99 for each birth weight tier) on the screening test. Of these 514 infants, 21 died before retest from various causes, of which extreme prematurity was the most frequent (mean weight 1.413,4 ± 970,4) and 376 (73%) had normal 17-OHP levels on retest. The remaining 117 infants with suspected CAH at retest were examined by a pediatrician and underwent serum or dried blood measurement of 17-OHP. CAH diagnosis was confirmed in eight infants. One of them initiated treatment before the second sample collection. Clinical and laboratory assessment of the other 109 patients (0.1% of the total population) was negative, and the patients were considered to be FP.

The estimated positive predictive value (PPV) of the initial screening test was 1.6%. Table 1 shows the rates of altered 17-OHP values at the initial screening according to birth weight tier in the general population screened until 40 days of age.

**Table 1:** Rate of altered 17-OHP results on initial CAH screening^a^ stratified by birth weight tier in the general population tested until 40 days of age in the state of Rio Grande do Sul, Brazil

Birth weight tier	Number	17 OHP (>P99 or two times P99)
		*n* (%)
≤1500 g	1071	35 (3.3%)
1501–2000 g	1773	71 (4.0%)
2001–2500 g	6462	106 (1.6%)
≥2501 g	95,431	302 (0.3%)
Total	104,737	514 (0.5%)

^a^17-OHP diagnostic cut-off levels: birth weight ≤ 1500 g: 110.4 ng/mL; birth weight 1501 to 2000 g: 43.0 ng/mL; birth weight 2001 to 2500 g: 28.2 ng/mL; and birth weight weight > 2500 g: 15.1 ng/mL

Median age was similar for CAH cases and false positive at the initial screening (*n* = 493) [8 (4.25–15.75) and 5 (4.0–6.0) days *P* = 0.199] and at retest (*n* = 492) [20.0 (17–20.0) and 17 (14–21) days, *P* = 0.205]. Median 17-OHP values at initial screening were significantly different between CAH cases and false positive [446.50 ng/mL (72.60–501.25) and 25.80 (17.4–41.8) ng/mL; *p* = 0.001]. The same was true for the retest, with a 17-OHP median of 435 ng/mL (209–521) and 8.30 (5.86–12.60) ng/mL (*p* < 0.001) respectively.

Table 2 shows 17-OHP values at the initial screening and retest according to birth weight tier. At the initial screening test as wells as at the retest, 17-OHP values were progressively lower with increasing weight. Delta 17-OHP levels (retest minus screening value) were also significantly different in each tier compared to the others.

**Table 2:** Median 17-OHP levels in infants with suspected congenital adrenal hyperplasia on newborn screening and retest according to birth weight tier

Sample^#^	Birth weight tier				*p*
	≤1500 g *n* = 23	1501–2000 g *n* = 67	2001–2500 g *n* = 105	≥2501 g *n* = 298 ^§^	
Screening (median ng/mL [P25-75])	154 (120 to 208)^a^	53.6 (47.0 to 64.7)^b^	33.6 (29.9 to 41.9)^c^	18.8 (16.0 to 23.4)^d^	<0.001
Retest (median ng/mL [P25-75])	48.1 (21.9 to 96.5)^a^	12.7 (10.1 to 20.5)^b^	8.1 (6.4 to 12.3)^c^	7.3 (5.1 to 10.6)^d^	<0.001
∆ Samples	−98.6 (−172.5 to −67.0)^a^	−38.9 (−47.2 to −33.2)^b^	−24.9 (−31.5 to −21.2)^c^	−11.8 (−15.6 to −7.95)^d^	<0.001

^#^17-OHP diagnostic cut-off levels: birth weight ≤ 1500 g: 110.4 ng/mL; birth weight 1501 to 2000 g: 43 ng/mL; birth weight 2001 to 2500 g: 28.2 ng/mL; and birth weight weight > 2500 g: 15.1 ng/mL; ^§^ *n* = 297 on retest

∆ Samples: difference between 17-OHP at retest and screening

Values are expressed as median and interquartile range; different superscript letters indicate statistical difference by GEE test

Regarding the 117 infants who underwent further clinical and laboratory evaluation of CAH, 61.5% (*n* = 72) were in birth weight tier 4 (>2500 g), vs. 7.7% (*n* = 9) in tier 1, 13.6% (*n* = 16) in tier 2, and 17% (*n* = 20) in tier 3. No CAH case was diagnosed in tier 1 or 2, with birth weight < 2000 g. The prevalence of maternal complications, such as gestational diabetes, maternal hypertension, or maternal infection was similar in the case and false positive groups. The frequency of neonatal complications (hypoglycemia, jaundice, sepsis, ventilation, oxygen therapy, diarrhea, vomiting) was also similar between these two groups. Comparison of the clinical and laboratory data obtained for cases and babies with false positive results are presented in Table 3. Significant differences were observed between the groups, with higher prematurity rate, lower gestational age, and lower weight in false positive patients. In turn, consanguinity and dehydration were more frequent in CAH cases. Also, lower levels of sodium, higher levels of potassium and higher serum levels of 17-OHP were detected in CAH patients, as was to be expected.

**Table 3:** Family history, maternal, perinatal, newborn and laboratory data of newborns diagnosed with congenital adrenal hyperplasia vs. false positive newborns

Variables	CAH cases (*n* = 8)	False positives (*n* = 109)	*p*
Maternal data			
Caesarean delivery (*n* [%])	4/8 (50.0)	42/70 (60.0)	0.496
Newborn data			
ICU care (*n* [%])	4/8 (50.0)	71/98 (72.4)	0.281
Preterm (*n* [%])	2/8 (25.0)	59/109 (54.1)	0.004
Birth weight (*n* [%])	2940 ± 570.34 (*n* = 8)	2496 ± 761.63 (*n* = 109)	0.110
Gestational age (week)	38.0 ± 1.9 (*n* = 8)	34.8 ± 3.2 (*n* = 72)	0.007
Dehydration (*n* [%])	5/8 (62.5)	3/76 (3.9)	<0.001
Na (nmol/L)^a^	122.25 ± 10.15 (*n* = 8)	136.56 ± 2.28 (*n* = 54)	0.005
K (nmol/L)^a^	6.17 ± 1.21 (*n* = 8)	5.31 ± 0.67 (*n* = 54)	0.004
Serum 17-OHP (ng/mL) (Md [P25-P75])	25.6 (12.8–285) (*n* = 3)	12.5 (7.4–17.8) (*n* = 45)	0.006
Family data			
Family history (*n* [%])	3/8 (37.5)	9/67 (13.4)	0.196
Consanguinity (*n* [%])	2/8 (25.0)	0/109 (0%)	<0.001

*CAH* Congenital adrenal hyperplasia, *ICU* Intensive care unit. Data are presented as percentage (Fisher’s exact test) or ^a^mean ± SD (Student’s t test)

## Discussion

Early diagnosis of CAH is crucial to prevent infant death due to adrenal insufficiency. In the present study, the first year of a CAH screening program provided by the public health care system in the state of Rio Grande do Sul, Brazil was assessed. The program successfully screened a high proportion of newborns (98.4%) between the 2nd and 40th post-natal days, and 80% of the valid samples were screened at the ideal moment, that is, between the 3rd and 7th post-natal days [2, 4, 10].

The incidence of CAH in the state of Rio Grande do Sul detected by the screening program, 1:13,551, was similar to that reported for other populations [1]. It was also very close to the incidence of 1:14,972 reported for the only adjacent Brazilian state, in which a similar, predominantly Caucasian population is found [19]. In contrast, other Brazilian states had a lower incidence of CAH [16, 17]. Ethnicity and geographic factors are known to affect the incidence of CAH [1, 6]. Thus, in a country such as Brazil, covering a large territory, with a racially mixed population, different ratios are to be expected. According to the latest Brazilian census, of 2010, 78% of the population in the South is white, in contrast to 42% in the Midwest and 55% in the Southeast [21]. Regarding confirmed CAH cases, the inability to diagnose the disease even in the presence of genital atypia has been reported in other Brazilian studies [10], and reinforces the need for universal newborn screening for CAH in Brazil. In this sense, improving time to test, transport time to the laboratory, and time to result is still a challenge that must be overcome. In turn, the 15 day-interval to retest seems to be adequate in most cases, since these are premature newborns, hospitalized in intensive care units, born from mothers who may have received corticoids during the final pregnancy days for improving fetal lung maturation.

Since 1977, when Pang et al. [22] described a microfilter paper assay for determination of 17-OHP levels in newborns, neonatal screening has been available for CAH due to 21-hydroxylase deficiency. Later, an immunofluorimetric assay was introduced, which is currently the most widely used technique worldwide [2, 23, 24]. More recent studies suggest a higher specificity and better sensitivity for mass spectrometry, especially when used as a second tier test [25–27]. In contrast, immunofluorimetric methods are less expensive, require a smaller blood spot, and are still widely available and recommended [2, 10, 24]. Also, mass spectrometry does not completely eliminate false positive results, especially in preterm infants [14, 27] .

In our sample, PPV (1.6%) and false positive rate were similar to those of previous reports [10, 24, 28]. False positive results are a long-standing concern of CAH neonatal screening programs [7, 9–11, 23, 27, 29]. In the past two decades, a decrease in false positive rates has been noted [10, 11, 23, 29, 30], possibly as a result of both improved 17-OHP detection methods and adjustment of diagnostic cut-off points to birth weight [7, 10]. Adjustment of diagnostic levels of 17-OHP according to birth weight tiers [7, 9, 10, 19] has been proposed as a useful strategy to minimize false positive. However, it is also important to recognize other possible factors associated with an increased 17-OHP level in newborns. Indeed, studies have shown that low birth weight, premature or critically ill infants may have elevated 17-OHP levels per se, without a link to 21-hydroxylase deficiency [8, 12, 31]. Possible explanations for the transient elevation in 17-OHP levels in these patients are immature hepatic function, leading to a decrease in the metabolic clearance of 17-OHP; increase in stress-induced production of 17-OHP, especially if the sample is collected in the first 24 h of life; or immaturity of the adrenal glands [31, 32]. Low birth weight, premature, and critically ill infants should be monitored in relation to 17-OHP concentrations, with a second sample collected on a later occasion to prevent false diagnoses and waste of resources.

We found an association between low birth weight and false positive results. The highest rate of false positive (4.0%) was found in the group with birth weight of 1500–2000 g (tier 2), in which no cases of CAH were finally detected (Table 1). We speculate that survival is more likely in tier 2 newborns as compared to those in tier 1 (<1500 g). We also recorded a higher rate of false positive results in preterm versus term infants (Table 3). Moreover, the gestational age of false positive babies was significantly lower than that of CAH cases. While a high correlation exists between birth weight and gestational age, one study suggests that gestational age-related 17-OHP cutoff levels improve CAH screening [9]. Nevertheless, birth weight data is more easily assessed than gestational age. Coulm et al. reported a PPV of 0.4% for CAH screening in pre-term infants, a value that is lower than that observed for term infants. Another study [33] suggests a correction factor for prematurity and weight, but does not use stratified cut-offs, which complicates the analysis of PPV. Interestingly, we observed that even if above the diagnostic cut-off point for the birth weight tier, 17-OHP values of false positive infants were significantly lower than those of CAH cases in both the initial screening and retest. Other studies have reported similar findings [10, 19], which might be explained by a more severe clinical status, since many of these false positive infants required intensive care [12].

Consanguinity was an important factor in this population, present in 25% of CAH cases but absent in false positive cases. Thus, adding information about consanguinity to the initial screening might support CAH diagnosis in the presence of high 17-OHP levels. Limitations of this study are its retrospective nature, which prevented the analysis of factors related to false positive results, and the lack of proper information on initial screening regarding prenatal use of glucocorticoid, which might affect 17-OHP levels. Prospective studies with adequate design are required for these analyses.

## Conclusion

The screening of CAH remains a challenge, and the implementation of an adequate screening flow makes population programs more assertive. In addition to the 17-OHP dosing method, diagnostic 17-OHP cut-offs stratified by birth weight, collection of samples at specific time points, and performance of retests even in the absence of clinical suspicion of CAH or confounding factors, such as prematurity and critical illness, greatly contribute to decrease false positive rates.

The present results support the need for CAH screening by the public health care system, and show that the strategy adopted is adequate, despite the initial screening of some infants after the 7th post-natal day. Future prospective studies may be useful to establish specific strategies for preterm groups, lower weight newborns, and ICU patients, and to improve effectiveness and PPV in all weight tiers.

## Abbreviations

17-OHP: 17 hydroxyprogesterone
CAH: Congenital adrenal hyperplasia
NC CAH: Non-classic congenital adrenal hyperplasia
PPV: Positive predictive value
SV CAH: Simple virilizing congenital adrenal hyperplasia
SW CAH: Salt-wasting congenital adrenal hyperplasia
